# Public Awareness and Perceptions of Longevity Determinants in Saudi Arabia: A Cross-Sectional Study (2024–2025)

**DOI:** 10.3390/healthcare13111229

**Published:** 2025-05-23

**Authors:** Lamah Allehaibi, Lamia Abuhaimed, Bashaer Hakami, Ayman Alotaibi, Sarah Alabbasi, Zain Alsharif, Taif Alayyafi, Asayel Alamri, Rawan Althaqil, Mohammed Alnuhait

**Affiliations:** 1 Pharmaceutical Practices Department, College of Pharmacy, Umm Al-Qura University, Makkah 21955, Saudi Arabia; 2 College of Pharmacy, Princess Nourah bint Abdulrahman University, P.O. Box 84428, Riyadh 11671, Saudi Arabia; 3 College of Pharmacy, King Saud bin Abdulaziz University for Health Sciences, P.O. Box 3660, Riyadh 11481, Saudi Arabia; 4 College of Medicine, Imam Mohammad Ibn Saud Islamic University (IMSIU), Riyadh 13317, Saudi Arabia

**Keywords:** longevity, public awareness, lifestyle factors, Saudi Arabia, healthy aging

## Abstract

**Introduction**: Longevity is increasingly recognized as the result of modifiable lifestyle, environmental, and social factors rather than genetics alone. While global interest in healthy aging is growing, public awareness of these determinants remains understudied in the Middle East. This study aimed to assess public awareness and perceptions of longevity-related factors among adults in Saudi Arabia. **Methods**: A cross-sectional online survey was conducted between November 2024 and January 2025 targeting residents of Saudi Arabia aged 18 and above. A researcher-validated questionnaire explored awareness across various domains, including lifestyle, environment, genetics, sleep, and religious beliefs. Perceptions were assessed using a series of items measuring beliefs about the influence of lifestyle, environmental, technological, and cultural factors on health and longevity. Descriptive and inferential statistics were used to analyze responses from 395 participants. **Results**: The sample included 395 participants, with 67.8% females and more than half (51.4%) aged 18–25 years. The participants exhibited high awareness of key lifestyle factors; greater than 88% acknowledged the role of sleep, and more than 90% recognized the importance of exercise and nutrition. However, fewer were aware of environmental (72.2%) or social determinants, and nearly half believed that genetic factors exert a primary influence on longevity. Higher awareness was significantly associated with behaviors like regular exercise (*p* = 0.004), dietary supplement use (*p* = 0.002), and recognition of the importance of sleep (*p* < 0.001). Younger adults showed higher awareness than older adults, while there were no significant differences in regards to gender, education, and income. Support for incorporating religious themes into health campaigns was also linked to greater awareness. Insurance status and chronic illness were not associated with awareness levels. **Conclusions**: While awareness of lifestyle-related longevity factors is strong, gaps remain in understanding broader influences such as environmental and social determinants. Culturally grounded public health strategies that integrate religious framing and emphasize actionable, evidence-based behaviors may enhance community engagement and support healthy aging across Saudi Arabia.

## 1. Introduction

Longevity, once viewed as a genetic endowment, is now increasingly understood as a multifactorial outcome shaped by behavioral, environmental, technological, and social influences. While genetics account for an estimated 20–30% of lifespan variability, the remainder is attributable to modifiable factors, such as physical activity, diet, social integration, and access to healthcare [1,2]. These findings have shifted the global health narrative from simply prolonging lifespan to optimizing healthspan, i.e., the number of years lived in good health without functional decline [3]. Although global life expectancy has steadily increased, many of the additional years are marked by poor health. From 2000 to 2019, life expectancy rose by more than six years, but the gains in healthy life expectancy were comparatively smaller [4]. This growing discrepancy raises critical questions about public understanding of the determinants of healthy aging and the role of education in bridging awareness gaps. Recent scientific progress in aging biology and technology has introduced promising interventions [5]. Artificial intelligence (AI) -driven diagnostics and personalized medicine have further contributed to the early detection and prevention of age-related diseases [6]. These advances are complemented by a growing body of research supporting behavioral interventions. For example, adherence to low-risk lifestyle factors, including regular physical activity, a balanced diet, and non-smoking, has been associated with an increase in life expectancy [7]. Psychosocial variables also exert a powerful influence. Strong social relationships have been associated with a greater likelihood of survival, while chronic loneliness increases the risk of mortality at levels comparable to those for smoking or obesity [8]. Mental well-being, community engagement, and cultural identity are increasingly being recognized as essential pillars of healthy aging. However, public awareness of these dimensions tends to be lower than that of diet and exercise, underscoring the need for more inclusive health education. Environmental exposures are another critical yet under-recognized factor. Air pollution, for instance, has been shown to shorten life expectancy by over two years globally [9]. However, public discourse often overlooks the role of environmental determinants in shaping long-term health outcomes, particularly in rapidly urbanizing regions. In Saudi Arabia, longevity research is gaining momentum. Life expectancy in the country has increased from less than 50 years in the mid-20th century to nearly 78 years at present [10]. Complementing this demographic shift is the emergence of the Hevolution Foundation, a Saudi-based nonprofit dedicated to funding global research on aging and promoting equitable access to healthy aging technologies. With a substantial commitment to geroscience, Hevolution aims to accelerate the translation of scientific discoveries into interventions that extend healthspan, positioning Saudi Arabia as a key contributor to the global longevity agenda [11]. At the same time, health communication has transformed. Social media, health apps, and online forums now serve as primary information sources for many individuals. While this digital transition improves access, it also exposes users to misinformation, including unsupported claims about supplements, diets, and anti-aging products [12]. Public misperceptions can lead to misplaced trust in unverified interventions and insufficient attention to well-established, evidence-based behaviors. Understanding what the public knows about longevity and what it overlooks is essential for designing targeted interventions. Most studies to date have focused on the clinical or biological aspects of aging, with limited attention paid to public awareness, especially in the Middle East. There is a pressing need to explore how individuals conceptualize longevity. Do they overestimate the role of genetics? Are they aware of the significance of social and environmental influences? How much confidence do they place in technological or religious approaches to aging? In this study, awareness refers to participants’ knowledge or recognition of various longevity-related determinants, such as lifestyle habits, environmental exposures, and health technologies. Perception refers to individuals’ beliefs, attitudes, and interpretations of how these factors influence lifespan and healthspan [13]. This study aims to evaluate public awareness of key longevity determinants, including genetic, behavioral, environmental, technological, and cultural, within the Saudi population. By identifying gaps and misconceptions, the findings can inform public health strategies and support national initiatives—such as those of Hevolution—which seek to enhance healthspan through science, education, and equitable policy.

## 2. Method

### 2.1. Study Design

This study employed a cross-sectional design using an online, self-administered survey to assess public awareness regarding key determinants of longevity. Data collection was conducted between November 2024 and January 2025 and targeted residents of Saudi Arabia. This study was designed to capture a broad representation of adults across various demographic backgrounds. The study population included individuals aged 18 years and older who were currently residing in Saudi Arabia and had access to the internet. Participation was voluntary, and informed consent was obtained electronically prior to survey initiation. The respondents were required to confirm their eligibility and consent to participate before proceeding to the questionnaire. The inclusion criteria were as follows: (1) Saudi and non-Saudi residents, (2) age ≥ 18 years, (3) access to an internet-enabled device, and (4) provision of informed consent. The exclusion criteria comprised the following: (1) individuals not residing in Saudi Arabia, (2) respondents younger than 18 years, (3) incomplete or inconsistent survey responses, and (4) lack of consent to participate. The survey instrument was developed in Arabic and distributed online. It was designed to be user-friendly and accessible across devices. The questionnaire covered sociodemographic characteristics, knowledge and perceptions related to longevity, lifestyle practices, and attitudes toward health-promoting behaviors. The content was informed by a review of the relevant literature and validated through expert review and pilot testing prior to deployment. The questionnaire was developed through an extensive literature review of studies related to public awareness of longevity determinants, followed by expert review by faculty members in pharmacy practice and public health to ensure content validity. A pilot test was conducted with 10 participants from the target population to refine its clarity, wording, and structure. Based on feedback, minor modifications were made before final distribution.

Responses were automatically recorded through the survey platform, de-identified, and exported to Microsoft Excel for organization and cleaning prior to statistical analysis. Ethical approval was obtained from the Institutional Review Board (IRB) at Umm Al-Qura University, and all procedures adhered to ethical guidelines for human subject research.

### 2.2. Sample Size

Given the limited availability of prior data on public awareness of longevity-related factors in Saudi Arabia, we assumed a conservative prevalence estimate of 50% awareness in the population. This approach maximizes sample size and ensures adequate precision. Using a 95% confidence level and a 5% margin of error, we applied a standard sample size calculation for proportions in large populations. With Saudi Arabia’s population estimated at approximately 36 million at the time of the study, the minimum required sample size was calculated to be 385 participants [14]. A convenience sampling approach was adopted, targeting adults aged 18 years and above residing in Saudi Arabia. This method was selected to enable broad participation across different regions and demographic backgrounds. The survey was disseminated electronically through widely used platforms to facilitate accessibility and capture diverse public perspectives.

The participants were eligible regardless of their prior knowledge about longevity, allowing the study to assess both awareness levels and general perceptions. All respondents were provided with an introductory statement explaining the purpose of the study, and participation was voluntary and anonymous. The average time required to complete the survey was approximately 10 min.

### 2.3. Questionnaire and Data Collection

Data were collected through an online self-administered questionnaire developed using Google Forms. The survey link was distributed via social media platforms and messaging applications to reach a wide cross-section of the Saudi population. The questionnaire was designed based on a comprehensive review of published literature on longevity determinants, public health awareness, and lifestyle-related behaviors [15,16]. Additionally, input was obtained from academic experts in pharmacy practice and public health to ensure that the content was relevant, clear, and aligned with the study objectives. To establish content validity and improve the clarity of items, the survey instrument underwent a pilot phase involving 10 participants representative of the target population. Their feedback led to modifications in the wording, formatting, and structure to enhance readability and comprehension. To assess internal consistency, the awareness score was evaluated using Cronbach’s alpha, which yielded a value of 0.82, indicating acceptable reliability. The finalized survey was available exclusively in Arabic to ensure cultural and linguistic appropriateness for the intended participants. It consisted of 44 items divided into five main sections. The first section gathered sociodemographic information, including age, gender, education, income, employment status, and region of residence. The second section assessed general health status, the presence of chronic conditions, health insurance coverage, and perceived barriers to adopting healthy lifestyle behaviors. Section three focused on awareness of factors affecting longevity, including medical advancements, environmental risks, lifestyle habits, and the role of technology. The fourth section explored cultural and religious beliefs related to health and aging, while the fifth examined lifestyle practices, such as dietary supplement use, physical activity, and sleep patterns. Additional items assessed sources of health information and preferences for future public health campaigns. The questionnaire was structured to take approximately 8–10 min to complete. Participation was entirely voluntary, and responses were anonymized to ensure confidentiality. To enhance data accuracy and quality, the survey platform was configured to allow only one response per device. Responses were monitored for completeness, and the instructions were clearly presented to the participants to ensure understanding. The instrument was also pilot-tested to improve its clarity and reduce ambiguity in regards to item wording. All collected data were stored securely and used solely for research purposes.

### 2.4. Statistical Analysis

All collected data were reviewed and cleaned for completeness and consistency prior to analysis. Responses with missing values or internal inconsistencies were excluded. Each variable was coded and anonymized to ensure confidentiality. Descriptive statistics are used to summarize participant characteristics. Categorical variables are expressed as frequencies and percentages, while continuous variables are presented as the means and standard deviations or medians and interquartile ranges, depending on data distribution. The Kolmogorov–Smirnov test was used to assess the normality of continuous variables. Non-parametric tests, including the Mann–Whitney U and Kruskal–Wallis tests, were applied for comparison of awareness scores across groups. Associations between awareness or practice levels and categorical sociodemographic and behavioral variables were assessed using Chi-squared tests. Where assumptions were not met, the Monte Carlo correction was applied to ensure accurate significance estimation. All statistical tests were two-tailed, with a *p*-value < 0.05 considered statistically significant. Analyses were performed using IBM SPSS Statistics version 25.0 (IBM Corp., Armonk, NY, USA).

## 3. Results

This study was carried out with 395 participants in Saudi Arabia. Table 1 summarizes the demographic characteristics among the participants. The studied participants primarily consisted of young adults aged 18–25 years (51.4%), with the majority being female (67.8%) and of Saudi nationality (96.7%). The largest portion of participants resided in the Western Province (67.6%), and most of them were single (57.5%). Their educational level was high, with 70.6% holding a bachelor’s degree. Their employment status varied, with 41.3% being students and 34.9% employed. In terms of income, the majority (40.8%) reported a monthly household income between approximately USD 1600 and 4000, while 31.9% earned less than USD 1600.

Additionally, 84.6% of participants considered their city to be urban, in terms of infrastructure and services [Table 1].

The general health status of the participants studied was largely positive, with 62.5% rating their health as excellent and 28.1% as very good, while a small percentage reported having good (7.8%) or poor (1.5%) health. Despite the overall positive health perception, 24.3% of participants reported having chronic diseases. Among these, hypertension was the most prevalent (8.6%), followed by diabetes mellitus (6.6%), thyroid diseases (4.6%), and psychological conditions, such as depression and anxiety (4.6%). Other chronic conditions included respiratory diseases, like tuberculosis (4.3%); obesity (4.1%); cardiovascular diseases (2.0%); irritable bowel syndrome (1.3%); and autoimmune diseases, such as systemic lupus erythematosus (1.3%). Less common conditions included cancer (0.5%), bone diseases (0.5%), polycystic ovary syndrome (0.3%), chronic kidney diseases (0.3%), seizures (0.3%), and chronic liver diseases (0.3%). Regarding health insurance coverage, a significant majority (71.1%) lacked health insurance, with only 28.9% having coverage. This highlights a potential gap in access to healthcare services among the participants. Financial status was also found to play a role in the ability to adopt healthy lifestyle practices. While 34.4% of participants believed that financial status did not affect their ability to maintain a healthy lifestyle, 40.0% stated that it affected them to some extent, and 25.6% believed it had a significant impact. These findings suggest that financial constraints and lack of health insurance could pose barriers to adopting and maintaining health-promoting behaviors, such as regular exercise and healthy eating, which are essential for longevity and overall well-being.

Table 2 illustrates the participants’ awareness and perceptions regarding factors influencing longevity.

Table 3 shows the participants’ perceptions of the role of religious practices in health and longevity. A significant majority (75.2%) strongly agreed that religious practices, such as prayer and fasting, contribute to better health and longer life, with an additional 17.2% agreeing. Only a small percentage (2.1%) disagreed. Engagement in religious activities was also high, with 84.1% practicing them daily and 5.6% weekly, while only 2.8% reported rarely or never participating.

**Figure 1:**
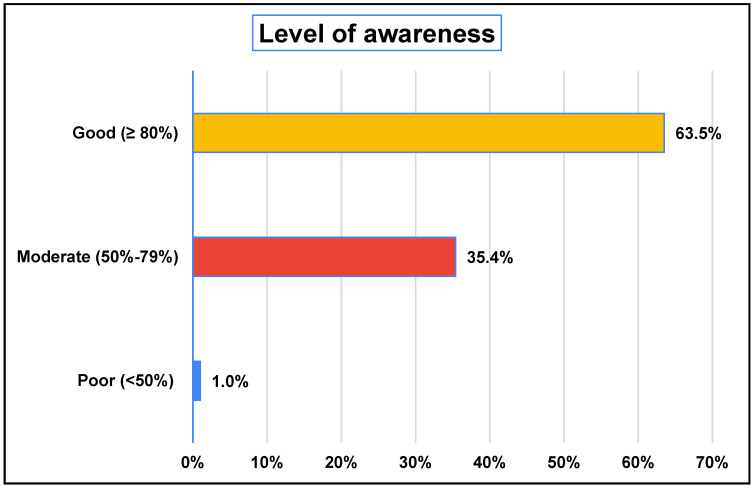
Distribution of the studied participants regarding level of awareness.

The awareness score was developed to quantitatively assess participants’ understanding of multiple determinants of longevity. It was composed of ten items covering key domains: medical advancements, environmental factors, social support, physical activity, interest in health-promoting practices, sleep, technology, religious beliefs, and cultural influences. Each item was scored based on predefined response scales, with binary items (e.g., “Yes/No”) scored as 1 or 0, and Likert-scale items ranging from 1 (e.g., “Strongly disagree” or “Not interested”) to 5 (e.g., “Strongly agree” or “Very interested”). One item assessing perceived understanding of social factors was scored from 1 to 3. The total awareness ranged from 6 to 30 points, with higher scores indicating greater awareness. To facilitate interpretation, raw scores were converted into percentages. Based on standard cutoff values, awareness levels were classified as poor (<50%), moderate (50–79%), or good (≥80%). This scoring approach allowed for a comprehensive and reliable evaluation of the participants’ awareness across diverse longevity-related dimensions and was supported by clear scoring logic and internal consistency in line with best practices for survey-based research. The awareness scores among the studied participants (N = 395) indicate a generally high level of awareness. The median awareness score was 25 (IQR: 22–27), with a mean of 24.31 ± 3.47, ranging from 6 to 30. When expressed as a percentage, the median awareness score was 83.3% (IQR: 73.3–90), with a mean of 81.05 ± 11.56, ranging from 20% to 100%. Regarding awareness levels, the majority (63.5%) demonstrated good awareness (≥80%), while 35.4% displayed a moderate level (50–79%). Only a small proportion (1.0%) exhibited poor awareness (<50%) [Figure 1].

Table 4 highlights the participants’ willingness to adopt healthier lifestyles, with 54.4% being very willing and 41.0% being somewhat willing to change their diet and exercise habits for better health and longevity. A total of 59.2% reported using dietary supplements or herbs to enhance their well-being, while 40.8% did not. The most commonly used supplements were vitamins, such as vitamin C or D (49.4%), followed by commercial herbal products (23.5%) and medicinal herbs (21.5%). Despite recognizing the importance of health, regular exercise was reported by only 37.2% of participants, while 62.8% did not engage in physical activity. Among those who exercised, walking or running was the most popular activity (53.9%), followed by strength training (19.0%) and aerobic exercises (13.2%). Additionally, most participants (95.9%) stated that insufficient sleep negatively impacts overall health and longevity. These findings suggest a need for greater encouragement of physical activity and structured health initiatives to translate willingness into action [Table 4].

The practice score was designed to reflect participants’ engagement in simple, evidence-based behaviors known to support longevity. It included three binary items: whether the participant currently uses dietary supplements or herbs believed to improve health, engages in regular physical exercise, and acknowledges the role of adequate sleep in supporting overall health and longevity. Each item was scored as 1 for “Yes” and 0 for “No”, producing a total score ranging from 0 to 3. To facilitate interpretation, the scores were also converted into percentages. The median practice score was 2 (IQR: 1–2), with a mean of 1.92 ± 0.77, ranging from 0 to 3. When expressed as a percentage, the median practice score was 66.7% (IQR: 33.3–66.7), with a mean of 64.14 ± 25.56, ranging from 0% to 100%. In terms of practice levels, 44.6% of participants demonstrated a moderate level (50–79%), while 24.6% exhibited good practice (≥80%). However, a significant proportion (30.9%) exhibited poor practice (<50%).

**Figure 2:**
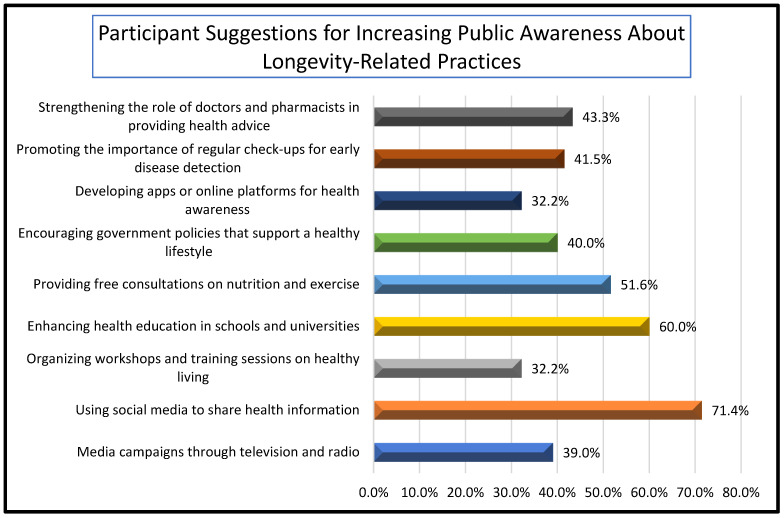
Participant suggestions for increasing public awareness about longevity-related practices.

The participants identified lifestyle choices (84.3%) and mental health (75.2%) as the most influential factors affecting longevity, followed by genetic predisposition (48.4%). Environmental factors (36.2%), religious practices (29.1%), and medical advancements (25.1%) were also acknowledged, although to a lesser extent. As illustrated in Figure 2, when asked about strategies to enhance public awareness of longevity-related practices, the majority favored the use of social media platforms (71.4%). This was followed by integrating health education into schools and universities (60.0%) and providing free consultations regarding nutrition and exercise (51.6%). Additional recommendations included conducting media campaigns (39.0%), promoting regular health check-ups (41.5%), implementing supportive government policies (40.0%), strengthening the role of healthcare professionals (43.3%), and developing digital health platforms (32.2%). These findings reflect a strong interest in both personal and system-level approaches to improving long-term health outcomes.

The analysis of factors associated with awareness levels among the study participants showed that age was significantly associated with awareness levels (*p* = 0.024), with younger participants (18–25 years) reporting a higher proportion of good awareness (50.6%), whereas older participants (46–55 years) showed a higher percentage of poor awareness (75.0%). Gender, nationality, region, marital status, educational level, employment status, household income, and urban residency did not show statistically significant associations with awareness levels (*p* > 0.05). The analysis of factors associated with practice levels among the studied participants showed no statistically significant associations between level of practice with age, gender, nationality, region, marital status, educational level, employment status, income, or urban residency (*p* > 0.05). There were significant associations between awareness levels and lifestyle factors, such as regular exercise (*p* = 0.004), dietary supplement use (*p* = 0.002), and the belief that lack of sleep affects overall health and longevity (*p* < 0.001), suggesting that individuals who engage in health-conscious behaviors tend to have higher awareness. Additionally, a strong association was observed between awareness and the belief that incorporating religious aspects into public health campaigns can enhance their effectiveness (*p* < 0.001). In contrast, no statistically significant associations were found between awareness levels and the presence of chronic diseases or health insurance coverage (*p* > 0.05).

## 4. Discussion

This study sheds light on the level of public awareness regarding longevity determinants within the Saudi Arabian context. The findings reveal that participants are generally well informed about lifestyle-related factors, such as regular physical activity, balanced nutrition, and adequate sleep, which have long been supported by the international literature as pillars of healthy aging [17]. However, the awareness of broader and less visible determinants, such as environmental influences, social connectedness, and the relative contribution of genetics, was noticeably more limited. Approximately half of the participants believed that genetics play the most significant role in determining lifespan. While genetic factors indeed contribute to longevity, their role is estimated to account for only 20–30% of its variance, with modifiable factors making up for the rest [1,18]. This overemphasis on heredity reflects a common misconception that may reduce motivation to adopt healthier behaviors. Religious and cultural influences were notably tied to participants’ perceptions of health and longevity. The overwhelming belief that religious practices, such as prayer and fasting, contribute positively to health aligns with studies linking spiritual engagement to enhanced well-being and longevity [19]. This finding also emphasizes the potential for faith-based public health messaging, particularly in a society where religion plays a central role in daily life. The positive association between support for religious framing in health campaigns and awareness scores further highlights this opportunity. Age was the most prominent demographic factor associated with awareness; younger adults (18–25 years) demonstrated higher awareness levels, likely reflecting increased digital literacy and access to online health content [20]. Conversely, variables such as gender, education level, and income did not significantly affect awareness, a finding that underscores the potential reach of digital platforms across social strata. Still, the association between higher awareness and engagement in health-promoting behaviors, such as regular exercise, supplement use, and sleep hygiene, suggests that those practicing healthier lifestyles are more informed or possibly more motivated to seek relevant knowledge. Interestingly, the absence of a significant difference in awareness among those with or without private health insurance or chronic conditions may reflect the accessibility and universality of healthcare services in Saudi Arabia. Public healthcare coverage ensures that most residents receive some level of health education and service access, regardless of insurance status, a strength of the national health system [11]. These results are supported by global evidence showing a strong public belief in lifestyle interventions for longevity [17]. This study adds to the relatively scarce body of research on how people in the Middle East understand and interpret factors that influence longevity. It offers insight into how cultural, religious, and social contexts shape public thinking about healthy aging. At a practical level, the results point to the importance of designing public health campaigns that do more than promote diet and exercise. Addressing overlooked areas, such as environmental influences and social well-being, could make these efforts more effective. Incorporating religious values and culturally relevant messaging may also improve the public’s connection to and trust in such initiatives, especially in Saudi Arabia. However, the relatively low awareness of environmental and social determinants is noteworthy. Air pollution, for example, has been shown to significantly reduce life expectancy [21]. Social isolation has been associated with mortality risk levels comparable to those for established clinical risk factors [22]. The fact that these areas are less understood among the public indicates a gap in public health messaging. The tendency to overvalue personal responsibility while overlooking structural and environmental influences has been identified in other studies as well [6]. Cultural parallels can be drawn between Saudi Arabia and other societies where religious and traditional values influence health decisions. The high daily engagement in religious activities reported by respondents reinforces findings that suggest a beneficial role of spirituality in health promotion. Faith-based campaigns have been shown to resonate well within Muslim communities, enhancing both awareness and behavior when designed in alignment with cultural and religious principles [23]. The high rate of dietary supplement use, despite limited evidence supporting their role in extending lifespan, raises important concerns. While supplements, such as vitamin D, may be necessary in deficiency cases, their widespread use “for longevity” is often driven more by marketing than by science [24]. This trend may also reflect the growing influence of unregulated health content online, especially among younger adults. Studies have noted that health misinformation on social media is widespread and may distort public understanding of effective interventions [24,25]. Our findings underscore the importance of targeted educational efforts to promote critical evaluation of health information, particularly on digital platforms. Public health efforts must aim to expand the public’s understanding of longevity beyond personal behaviors, incorporating social determinants and environmental exposures. Campaigns could, for instance, highlight the long-term effects of air pollution, poor urban infrastructure, and climate-related stressors, translating global evidence into local narratives [20]. In parallel, they should address the benefits of social connectedness, family engagement, and mental well-being, all of which are known to influence health outcomes. The role of healthcare providers and institutions is crucial in disseminating reliable information. Given the wide reach of Saudi Arabia’s public healthcare network, primary care centers, clinics, and hospitals can be leveraged as consistent touchpoints for educating patients from all backgrounds. Brochures, waiting room visuals, and brief consultations can reinforce messages about evidence-based longevity practices. Moreover, bridging the generational gap in health awareness should be a key component of future interventions. Older adults may require tailored educational methods that rely less on digital engagement and more on face-to-face interactions, community sessions, or national television campaigns [15]. Younger adults, while more digitally connected, should be guided toward verified sources and taught to critically assess the health content they encounter online. Programs such as the Hevolution Foundation and national initiatives focused on healthy aging can play a pivotal role in coordinating and funding these efforts [11]. They can support culturally adapted campaigns, fund longitudinal studies, and help develop digital health literacy programs aimed at countering misinformation. Beyond the local context, these findings may also offer insights applicable to other countries, particularly those in the Middle East and North Africa (MENA) region or settings where religious and cultural beliefs strongly shape public health behaviors. Understanding how these factors influence perceptions of longevity can support the design of culturally aligned health promotion efforts across diverse societies. This study also presents several strengths. It is among the first in Saudi Arabia to explore longevity awareness across lifestyle, cultural, and environmental domains. The sample size was robust, and the tool used showed good internal consistency. Due to the online distribution method, there is a possibility of selection bias favoring younger, educated, and digitally literate respondents, which may limit the generalizability of the findings to the wider population. Self-reported data also pose potential bias risks, especially around socially desirable behaviors, such as religious observance and healthy living. Furthermore, as a cross-sectional study, causal relationships cannot be established. Despite these limitations, this study contributes valuable data regarding public perceptions of longevity determinants and highlights areas to which education and policy can be directed. It sets the stage for further research that includes underrepresented populations, utilizes objective measures, and evaluates the impact of culturally adapted interventions.

## 5. Conclusions

This study revealed a high level of public awareness regarding core lifestyle behaviors that promote longevity among adults in Saudi Arabia, particularly concerning exercise, nutrition, and sleep. However, gaps remain in understanding the influence of environmental and social factors, alongside misconceptions about the dominant role of genetics. Cultural and religious beliefs were found to strongly shape perceptions, and younger individuals demonstrated greater awareness than their older counterparts. These findings underscore the importance of culturally tailored public health strategies that address both knowledge and behavior, with an emphasis on translating awareness into actionable, evidence-based practices. Health campaigns should use culturally relevant messages delivered through trusted community channels. Strengthening healthy aging will also require coordinated efforts across sectors, including collaboration between public health authorities, educational institutions, and environmental agencies to ensure consistent and reinforced messaging that resonates with the population. Policies should also give more attention to social support and environmental factors in regards to aging.

## Figures and Tables

**Table 1:** Socio-demographic characteristics of the participants.

Survey Items		Studied Participants (N = 395)	
		n	%
Age	18–25 years	203	51.4%
	26–35 years	57	14.4%
	36–45 years	56	14.2%
	46–55 years	52	13.2%
	>56 years	27	6.8%
Gender	Female	268	67.8%
	Male	127	32.2%
Nationality	Saudi	382	96.7%
	Non-Saudi	13	3.3%
Region	Central Province	45	11.4%
	Eastern Province	25	6.3%
	Northern Province	18	4.6%
	Southern Province	38	9.6%
	Western Province	267	67.6%
	Makkah Province	2	0.5%
Marital Status	Divorced	13	3.3%
	Married	152	38.5%
	Single	227	57.5%
	Widowed	3	0.8%
Educational Level	Primary	3	0.8%
	Intermediate	7	1.8%
	Secondary	69	17.5%
	Bachelor’s	279	70.6%
	Diploma	10	2.5%
	Master’s	22	5.6%
	Doctorate	5	1.3%
Employment Status	Employed	138	34.9%
	Unemployed	54	13.7%
	Business Owner	15	3.8%
	Student	163	41.3%
	Retired	25	6.3%
Monthly Household Income	USD 4000–8000	81	20.5%
	USD 8000–13,300	19	4.8%
	USD 1600–4000	161	40.8%
	Less than ~USD 1600	126	31.9%
	More than USD 13,300	8	2.0%
Perceived Urban Classification of Current Residence	Yes	334	84.6%

**Table 2:** Awareness and perceptions of longevity-related factors among the study participants.

Survey Items		Study Participants (N = 395)	
		n	%
Awareness of medical advancements related to longevity	No	190	48.1%
	Yes	205	51.9%
Primary source of information on longevity-related medical advancements *	Healthcare practitioners	79	20.0%
	Traditional media (TV, newspapers)	83	21.0%
	Social media	152	38.5%
	Family or friends	50	12.7%
	Other: University	5	1.3%
	Not previously aware of longevity-related medical advancements.	174	44.1%
Awareness of the impact of environmental factors (e.g., pollution, climate change) on longevity	No	110	27.8%
	Yes	285	72.2%
Level of understanding of how social factors (e.g., support networks) influence longevity	Moderate understanding	182	46.1%
	High understanding	159	40.3%
	Unaware	54	13.7%
Perception of the role of regular physical activity in promoting longevity	Strongly agree	237	60.0%
	Agree	127	32.2%
	Neutral	24	6.1%
	Disagree	6	1.5%
	Strongly disagree	1	0.3%
Level of interest in learning about longevity-promoting practices	Not interested	85	21.5%
	Interested	182	46.1%
	Very interested	128	32.4%
Awareness of the role of adequate sleep in promoting health and longevity	No	45	11.4%
	Yes	350	88.6%
Perception of technology (e.g., health apps) in supporting longevity and health	Strongly agree	92	23.3%
	Agree	145	36.7%
	Neutral	121	30.6%
	Disagree	29	7.3%
	Strongly disagree	8	2.0%

* More than one option was selected.

**Table 3:** Religious and cultural aspects among the studied participants.

Survey Items	Study Participants (N = 395)	
	n	%
Perception that religious practices (e.g., prayer, fasting) contribute to health and longevity		
Strongly agree	297	75.2%
Agree	68	17.2%
Neutral	22	5.6%
Disagree	7	1.8%
Strongly disagree	1	0.3%
Frequency of engagement in religious practices that support mental and physical health		
Daily	332	84.1%
Weekly	22	5.6%
Sometimes	30	7.6%
Rarely	6	1.5%
Never	5	1.3%
Belief that local culture promotes healthy lifestyles linked to longevity		
No	187	47.3%
Yes	208	52.7%
Perception that incorporating religious elements into public health campaigns enhances effectiveness		
Strongly agree	225	57.0%
Agree	122	30.9%
Neutral	38	9.6%
Disagree	6	1.5%
Strongly disagree	4	1.0%

**Table 4:** Lifestyle and longevity practices among adults in Saudi Arabia.

Survey Item	Study Participants (N = 395)	
	n	%
Willingness to change lifestyle (e.g., diet, exercise) to improve health and longevity		
Not willing	18	4.6%
Somewhat willing	162	41.0%
Very willing	215	54.4%
Use of dietary supplements or herbs believed to improve overall health		
No	161	40.8%
Yes	234	59.2%
Types of supplements/herbs used (multiple responses allowed)		
Vitamin supplements (such as vitamin C or D)	195	49.4%
Mineral supplements (such as calcium or magnesium)	76	19.2%
Protein supplements (such as whey protein)	22	5.6%
Fish oil or omega-3 supplements	79	20.0%
Medicinal herbs (such as fenugreek, ginger, or turmeric)	85	21.5%
Commercial herbal products (such as black seed or honey-based products)	93	23.5%
Energy or endurance supplements (such as caffeine or sports supplements)	62	15.7%
Regular engagement in physical exercise		
No	248	62.8%
Yes	147	37.2%
Types of physical activity practiced (among those who exercise)		
Walking or running	213	53.9%
Strength training (weightlifting)	75	19.0%
Yoga or meditation exercises	39	9.9%
Aerobic exercises (such as Zumba or cardio)	52	13.2%
Team sports (such as football or basketball)	26	6.6%
Swimming	27	6.8%
Cycling	18	4.6%
Perception that inadequate sleep negatively affects health and longevity		
No	16	4.1%
Yes	379	95.9%

## Data Availability

The data supporting the findings of this study are available from the corresponding author upon reasonable request. Due to the inclusion of personal and regional identifiers, restrictions apply to protect participant confidentiality.
